# Supplementary material for: Challenges to diagnosing and managing preeclampsia in a low-resource setting: A qualitative study of obstetric provider perspectives from Ghana
Source: PLOS Glob Public Health. 2023 May 2;3(5):e0001790. doi: 10.1371/journal.pgph.0001790 (PMC10153692; doi:10.1371/journal.pgph.0001790)
Supplement: S1 File — (DOCX) [file pgph.0001790.s001.docx]

**Interview Guide:**

**Provider Perspectives on Challenges Diagnosing and Managing Preeclampsia**

*Hello. Thank you for agreeing to participate in our study about provider perspectives on challenges diagnosing and managing preeclampsia.*

*There are a few things I will go over with you before starting the interview. First and foremost, this interview is completely confidential. This means I cannot tell anyone about your answers. In addition, your name will not be associated with your responses. Instead, we will assign you a study number so that only those of us running the study know that these are your responses. Please feel free to be honest and open about your experience, as there will be no consequences. If there are any questions that make you uncomfortable or you wish to stop the interview, you can let me know at any point. And lastly, feel free to ask me any clarifying questions if necessary.*

*The interviews include open-ended questions about your experiences diagnosing and managing preeclampsia. Interviews are expected to last approximately 30 minutes. The interview portion will be audio-recorded for later transcription and analysis. The recording will be stored securely. After they are transcribed, the recordings will be deleted. The recordings and transcriptions will not be linked to your name or any other identifying information.*

*Before signing the consent form, do you have any questions?*

***[sign consent form]***

*I will turn on the recorder now so I can record our discussion. For these questions, the answers can be as long as needed for you to answer them fully.*

Section 1: Preeclampsia in Ghana

First, I would like to ask about your experiences managing preeclampsia as a doctor in Ghana, and the challenges you may face.

1. Tell me about your experience with preeclampsia.
   1. How common is preeclampsia amongst your patients?
      1. Why do you think it is so common/uncommon?
   2. How do your patients usually first present with preeclampsia? (i.e. are they diagnosed in a clinic or present while in labor or recognize warning symptoms at home?)
      1. Do they present initially with symptoms or develop them later on?
   3. What are typical health outcomes in your patients with preeclampsia? (i.e. do they develop complications, or usually recover quickly?)
      1. Why?
2. Do your patients with preeclampsia usually present early or late (with complications)?
   1. Why?
3. In your experience, what are the biggest challenges to diagnosing preeclampsia early?
   - 1. Health system factors?
        1. Specific Prompts
           1. High patient to provider ratio, inadequate patient counseling, no phone triage system, poor health education, etc.
     2. Individual patient factors?
        1. Specific Prompts
           1. Patient interest, agency, capability, health literacy, etc.
4. Tell me about the challenges you face in providing good preeclampsia care to your patients.
   1. Specific Prompts
      1. Do you have the time you need?
      2. Do you have the resources you need?
      3. Do you face issues with the health system?
      4. Do you face issues with patients?
      5. What are other barriers?
5. What solutions/ interventions do you think might be helpful to improve preeclampsia care in your practice?
6. What solutions/interventions do you think might be helpful to diagnose preeclampsia earlier (before complications develop)?

Is there anything else you would like to share about your experiences caring for women with preeclampsia?

Section 2: Demographics

**Clinical Role:**

House Officer

Junior Resident in Obstetrics/Gynaecology

Senior Resident in Obstetrics/Gynaecology

Consultant in Obstetrics/Gynaecology

**Gender:**

Male

Female

Other/ Prefer Not To Respond

**Years in practice as a doctor:**

<1 year

1-5 years

6-10 years

11-20

>20 years

**Average Number of Patients with Preeclampsia Managed Weekly:**

0-5

6-10

11-15

16-20

>20

**Average Number of Patients with Eclampsia Managed Weekly:**

0

1

2

3

4

5

**Closing:**

“*We have now completed the interview. Thank you so much for your time and for participating. Is there anything you have questions or comments on?*
