# Supplementary material for: Challenges to diagnosing and managing preeclampsia in a low-resource setting: A qualitative study of obstetric provider perspectives from Ghana
Source: PLOS Glob Public Health. 2023 May 2;3(5):e0001790. doi: 10.1371/journal.pgph.0001790 (PMC10153692; doi:10.1371/journal.pgph.0001790)
Supplement: Attachment — Submitted filename: Response to Reviewers.docx [file pgph.0001790.s003.docx]

January 13, 2022

Submission ID: PGPH-D-22-01570

PLOS Global Public Health

Patrick A. Palmieri, Academic Editor

Dear Patrick A. Palmieri,

Thank you very much for the opportunity to revise our manuscript: "Challenges diagnosing and managing preeclampsia in a low-resource setting: A qualitative study of obstetric provider perspectives from Ghana,” which is now titled “*Challenges to diagnosing and managing preeclampsia in a low-resource setting: A qualitative study of obstetric provider perspectives from Ghana*.” We appreciate the comprehensive and thoughtful reviews. We have incorporated the reviewers’ comments and believe that the manuscript is now much stronger.

Below, we address each point raised by the reviewers. We have uploaded a marked-up copy of the manuscript with tracked changes (highlighted in yellow) made to the original version as well as an unmarked copy.

Sincerely,

Titus Beyuo, on behalf of all authors

Reviewer #1 Comments

**Title Comments**

**1. Preposition missing in the title …... Challenges ? diagnosing and managing…**

**Response:** Thank you for raising this point. We agree that a preposition was missing and have changed the title to ensure better clarity. It is now titled “*Challenges to diagnosing and managing preeclampsia in a low-resource setting: A qualitative study of obstetric provider perspectives from Ghana*” (Line 1).

**2. Can this sentence be rewritten to make more sense. The “but” in this sentence should be removed “The greatest burden of disease is in low- and middle-income countries where healthcare providers face significant, but understudied, challenges to diagnosing and managing preeclampsia”**

**Response:** Thank you for raising this point. We agree that this sentence makes more sense without the “but.” It is now removed in the manuscript (Lines 29-31).

**Methods**

**3. You have stated that “Participants’ thoughts and opinions were gathered without the bias of researchers’ assumptions about preeclampsia care in Ghana.” Can you justify how this being done. Did you engage bracketing in the data collection and analysis process to ensure that any preconception notions from researchers do not taint the process ?**

**Response:** Thank you for raising this point. Our approach to limiting researchers’ bias included purposefully creating a semi-structured interview guide with very broad, open-ended questions to encourage participants to share any thoughts or opinions that come to their mind. Additionally, the primary researcher conducting interviews was intentionally chosen to be an objective member with no prior experience working within the Ghanaian healthcare system in order to limit the impact of preconceived notions, if any. We agree that this was not as clear in the current manuscript, so we added the following text: *“The researchers’ bias was limited by purposefully creating a semi-structured interview guide with very broad, open-ended questions to encourage participants to share any thoughts or opinions that come to their mind. In addition, the primary researcher conducting interviews was intentionally chosen to be someone without prior experience working within the Ghanaian healthcare system.”* (Lines 161-165).

To clarify that the study and data analysis, however, were conducted with the input from senior research team members who have more experience with the Ghanaian healthcare system, we added the following sections of text: *“All interviews were conducted by a non-Ghanaian female medical student to ensure objectivity and limit the impact of preconceived notions. She received training on qualitative interviewing techniques and cultural humility by senior members of the research team who have significant experience with the Ghanaian healthcare system”* (Lines 167-171) and *“Transcripts were independently reviewed by two researchers (primary interviewer and senior research team member) who separately developed sets of key-word phrases”* (Lines 178-180).

**Discussion**

**4. You need to weave your study aims/objectives at the beginning of your discussion so that the reader knows you achieved them. This can be a sentence under “principal finding”**

**Response:** Thank you for raising this point. We agree that the first sentence in the discussion should have more clarity to show that we achieved our study aims. Based on the study aims identified in the introduction (Lines 96-98), we expanded the first sentence to be the following: *“Interviews with obstetric doctors at a tertiary care hospital in urban Ghana identified critical challenges faced at the patient, provider, and systemic level to detecting and managing preeclampsia, which mediate the health outcomes of a pregnancy complicated by preeclampsia”* (Lines 385-387).

**Strength and limitation**

**5. Is the first sentence a strength or limitation ? Interviewing highly trained and knowledgeable clinicians. Do the researchers ever thought that this could be biased as clinicians may talked based on their knowledge rather than their experiences, such disadvantages of the population.**

**Response:** Thank you for raising this point. We believed interviewing clinicians was a strength since our study aimed to understand diagnostic and management challenges, and clinicians are at the center of that and hold the most responsibility. This rationale is also explained in the introduction (Lines 98-101).

However, we do agree that clinicians may be biased and may not have direct experience of the challenges they identified at the patient-level. We added the following text to expand upon this limitation: *“Finally, while doctors identified challenges faced at the patient-level, they may not have direct experience or complete comprehension of the challenges patients face in accessing and adhering to recommended preeclampsia care. Therefore, future studies should also include patient perspectives”* (Lines 480-483).

Reviewer #2

**This manuscript discusses the challenges with diagnosing and managing preeclampsia within an urban, tertiary hospital in Accra, Ghana. The study is methodologically sound and produces interesting results that contribute to the literature on this topic. With a few minor changes, I believe this manuscript is appropriate for publication.**

**Response:** Thank you for this positive feedback.

**Introduction:**

**The authors give a good overview of preeclampsia and the complications that result from it. It would be helpful if the authors add a paragraph about the burden of preeclampsia specifically in Ghana and its implications. Have there been any efforts to address the issue in recent years?**

**Response:** Thank you for the positive feedback. We agree that more information about the burden of preeclampsia in Ghana would be helpful. We added the following text: *“In Ghana where this study is proposed, incidence of hypertensive disorders in pregnancy is estimated at 7.6%. Despite improvements in clinical protocols and increased access to key medications like antihypertensives and magnesium sulfate, rates of eclampsia continue to be significantly higher than in high-income countries, and neonatal outcomes are poor. Institutional reports from two major tertiary-level hospitals suggest that hypertensive disorders have overtaken hemorrhage as the leading cause of maternal mortality [7,8].”* (Lines 86-92).

**Methods:**

**The methods section is soundly written. It might be helpful to start out the section with your study design and then move into the study setting, etc. In terms of recruitment, how many total eligible participants were there? This would help explain some of the findings discussed in the results section (e.g., lack of sufficient healthcare personnel). Additionally, how was the interview guide developed? Did you use a theoretical framework or was it based on previous research? It might be helpful to include the interview guide as a supplemental file.**

**Response:** Thank you for this overall positive feedback.

The methods section was reorganized, as suggested.

In terms of recruitment, a total of 152 participants were eligible (Line 140).

The interview guide was developed using grounded theory with broad, open-ended questions to encourage participants to share any thoughts or opinions that come to their mind. This process is described in the methods section (Lines 156-165). We agree that providing the interview guide would be helpful, and so we have included it as a supplemental file.

**Results and Discussion:**

**The results and discussion are strong and do not require any substantial changes. I would make one remark in relation to the strengths and limitations section. The authors state that interviews were conducted by an American research team member to limit concerns with sharing challenges related to the hospital setting or clinical practice. I would caution away from framing this statement in this way and instead say the interviews were conducted by an objective member of the research team that is not affiliated with the hospital. There is a lot of hesitancy and reservations related to Americans going to LMICs and conducting research, so if you choose to include the statement as is, it would be important to also have a reflexivity statement to share your positionality.**

**Response:** Thank you for the positive feedback. We agree that the current framing of “American” research team member may invite scrutiny. Therefore, we changed the language to refer to the primary interviewer as a non-Ghanaian female medical student and included additional rationale to justify our choice (Lines 163-165, Lines 167-169, and Line 476).

Reviewer #3

**1. Important issue has been raised. It seems the condition of care is different between low income countries.**

**Response:** Thank you for the positive feedback.

**2. The study clearly shows the obstetric and gynecology service situation as well as religious and social life in Ghana.**

**Response:** Thank you for the positive feedback.

**3. I would recommend the researchers to take more sampling from other tertiary hospital or maternity hospitals which are providing preeclampsia and eclampsia care. Or to interview the midwives to ensure the study results.**

**Response:** Thank you for raising this point. We agree that future research studies are necessary to corroborate our study’s findings through both sampling of other hospitals (including non-tertiary) and increased diversity of participants including midwives and patients themselves. These points are illustrated in our strengths and limitations section (Lines 469-471, Lines 477-483). Our justification for choosing only a tertiary hospital for this initial study is also included in this section (Lines 466-469).
