# Supplementary material for: Challenges to diagnosing and managing preeclampsia in a low-resource setting: A qualitative study of obstetric provider perspectives from Ghana
Source: PLOS Glob Public Health. 2023 May 2;3(5):e0001790. doi: 10.1371/journal.pgph.0001790 (PMC10153692; doi:10.1371/journal.pgph.0001790)
Supplement: Attachment — Submitted filename: Response to Reviewers_2nd revisions.docx [file pgph.0001790.s004.docx]

February 1, 2023

Submission ID: PGPH-D-22-01570

PLOS Global Public Health

Patrick A. Palmieri, Academic Editor

Dear Patrick A. Palmieri,

Thank you very much for the opportunity to submit a minor revision for our manuscript: “*Challenges to diagnosing and managing preeclampsia in a low-resource setting: A qualitative study of obstetric provider perspectives from Ghana*.” We appreciate the comprehensive and thoughtful reviews.

Below, we address each point raised by the reviewers. We have uploaded a marked-up copy of the manuscript with tracked changes made to the original version as well as an unmarked copy.

Sincerely,

Titus Beyuo, on behalf of all authors

Reviewer #4:

**Comment**: In the section on the research design (lines 139-142), the authors state that grounded theory was used but the explanations of data collection, analysis, and the format of the results are not consistent with grounded theory. Reference to grounded theory should be removed.

**Response:** Thank you for raising this point. We agree and have removed the reference to grounded theory, as suggested.
